# All-carbon 3D-structured electrodes by direct ink writing as electrocatalyst supports for alkaline freshwater and seawater electrolysis

**DOI:** 10.1080/14686996.2026.2688057

**Published:** 2026-06-23

**Authors:** Lucía Muñiz Muñoz, María González-Ingelmo, Pablo Rodríguez Lagar, Miriam López García, Jonathan Ruiz Esquius, Daniel Barreda, Ricardo Santamaría, Clara Blanco, Victoria G. Rocha

**Affiliations:** INCAR-CSIC, Instituto de Ciencia y Tecnología del Carbono, Oviedo, Spain

**Keywords:** Electrocatalyst, graphite, direct ink writing, water splitting, seawater

## Abstract

Water splitting is a key technology for green hydrogen production, yet important challenges persist, particularly as large-scale expansion is expected, with seawater electrolysis emerging as a promising alternative to mitigate freshwater limitations. Among these challenges, the development of active, selective, and long-term stable electrocatalysts is crucial. The design of the catalyst support plays a pivotal role, and three-dimensional (3D) supports offer unique opportunities to enhance performance and optimize electrochemical reactor configurations. In this study, we demonstrate the use of 3D-structured all-carbon electrodes by Direct Ink Writing (DIW) as versatile supports for electrocatalyst preparation. NiCo-CeO_2_/3D-Gr and NiFe/3D-Gr were synthesized by electrodeposition and employed as hydrogen evolution reaction and oxygen evolution reaction catalysts, respectively. Both electrodes exhibited excellent electrocatalytic behavior in alkaline and alkaline-saline media. Overall water splitting was conducted at 100 mA cm^−2^ for 200 h, achieving voltages below 2 V and exceptional stability. This work highlights the potential of 3D-structured all-carbon electrodes by DIW as a platform for advanced electrocatalyst development, offering tunable architectures and a pathway for future optimization.

## IMPACT STATEMENT

This study presents 3D-structured all-carbon electrodes as robust and versatile platforms for integrating active electrocatalysts for water splitting, enabling stable performance in alkaline and challenging alkaline-saline environments.

## GRAPHICAL ABSTRACT

**Figure:**
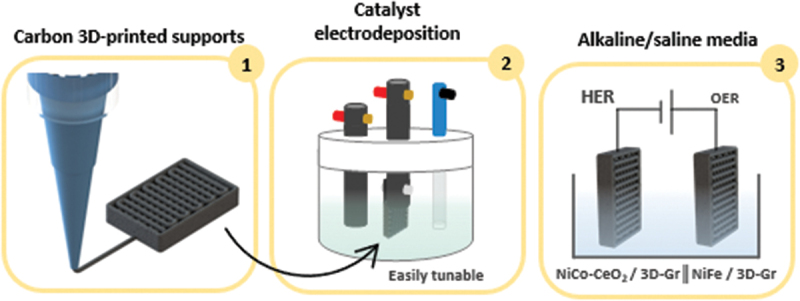

## 1. Introduction

Hydrogen produced via water electrolysis powered by renewable energies is known as green hydrogen. This process involves the hydrogen evolution reaction (HER) and oxygen evolution reaction (OER), and it is considered a key technology for the transition toward a carbon-neutral energy economy [1]. Thanks to the recent advances in electrocatalysts and overall electrolyzer technology, green hydrogen production is expected to significantly increase in the coming years (with capacity likely to grow from about 2 GW in 2024 to 70 GW by 2030) [2]. Since current water electrolysis technologies rely on freshwater [3], the projected expansion of green hydrogen raises concerns about its consumption, which is already limited in some arid regions. Consequently, seawater electrolysis has emerged as an attractive alternative since it constitutes approximately 96.5% of the world’s water supply [4]. However, operating under saline conditions introduces additional challenges, such as chloride-induced corrosion [5], the competitive chlorine evolution reaction (ClER), and electrode degradation [6], which require further research to develop advanced electrocatalysts with high activity, selectivity, and long-term stability. Novel strategies include electronic structure modulation, oxygen vacancy engineering, amorphous and porous architectures, corrosion-resistant coatings, and, importantly, the development of stable and conductive supports for the catalysts [7]. The latter is particularly crucial, as the effectiveness of most of the mentioned approaches depends on the stability and properties of the support, which ensures that the catalyst can remain active under electrocatalytic conditions [8].

In addition to chemical stability and conductivity, the architecture of the support plays a decisive role in the overall electrode performance. Three-dimensional (3D) structures [9,10] can facilitate electrolyte diffusion and gas bubble release [11,12], improving mass transport [13,14], and maintaining active surface exposure during operation [15]. Commonly used supports, such as nickel foam or carbon paper, exhibit good conductivity and mechanical strength; however, they are restricted to the composition, porosities, and geometries available commercially. Therefore, the development of alternative 3D supports with tunable composition and architecture represents an appealing route to optimize electrode design for water electrolysis applications.

Additive manufacturing (AM), also known as 3D printing (3DP) [16], offers a powerful approach to overcome these limitations by enabling the fabrication of electrodes with precise control over geometry, porosity and composition [17] at different length scales. Techniques such as fused deposition modelling (FDM), digital light processing (DLP) and selective laser melting (SLM) have been successfully employed for the preparation of electrodes for OER and HER, owing to their ability to produce mechanically robust and conductive metallic structures [18–21]. However, these AM technologies have limited compatibility with diverse materials and provide less flexibility in tuning porosity or composition [22]. In this regard, direct ink writing (DIW) is a particularly versatile AM technology, as it can process virtually any precursor that can be formulated as a paste with the suitable rheology, while offering a simple, low-cost, and rapid fabrication route [23,24]. In DIW, accessible filament dimensions and structural complexity are governed by the interplay between ink formulation and printing parameters. Tailored inks exhibiting shear-thinning behavior and a finite yield-stress enable stable extrusion and shape retention, while nozzle size, filament spacing, toolpath, and infill determine the final architecture and its porosity [25]. Most importantly, if previously reported catalyst preparation methods, such as hydrothermal synthesis, wet impregnation, or electrodeposition, are combined with these tailored 3D-structures, a promising field of investigation is opened, enabling a high degree of material versatility and the design of custom electrodes with optimized geometry and functionality. In particular, electrodeposition stands out as a simple, scalable, and easy-to-control technique for uniformly coating conductive 3D-printed supports with catalytic materials [26].

The authors have recently developed a graphite paste formulation which, when printed as a grid-like three-dimensional electrode by DIW [27], exhibits not only mechanical and electrical properties that surpass any other 3D-printed carbon electrodes [28,29], but also excellent electrochemical performance in vanadium redox flow batteries (RFB) compared with commercial carbon felts. The grid-like structure of the electrodes was shown to contribute directly to the improved battery performance in flow conditions, which was further enhanced by tuning the electrode composition, for example, through the incorporation of carbon nanotubes. The outstanding mechanical and electrical properties of these all-carbon DIW structures, where the final electrode is graphitic unlike other common AM electrodes such as FDM or SLM [30,31] that generally contain non-conductive components, make them promising candidates as catalyst supports in electrochemical flow reactors (RFB, fuel cells, electrolysers, etc). These technologies are involved in the large-scale integration of intermittent renewable energy sources and traditionally rely on 3D structures such as commercial metallic foams or carbon fiber papers/felts.

Herein, we present a proof-of-concept for the use of 3D-structured all-carbon electrodes fabricated by DIW, optimized from those previously reported by our group [27], as novel supports for electrocatalysts. From the wide range of non-noble metal catalysts reported to date, NiCo-CeO_2_ [32] and NiFe [33] catalysts were selected due to their excellent performance toward the hydrogen evolution reaction (HER) and oxygen evolution reaction (OER), respectively, and their suitability for simple electrodeposition-based synthesis onto the all-carbon DIW electrodes. Their activity and stability were evaluated under alkaline and alkaline–saline conditions, demonstrating the feasibility of DIW-printed graphite architectures as robust and tunable supports. This study establishes the basis for future developments of advanced 3D electrode designs tailored to demanding electrochemical processes such as seawater splitting.

## 2. Experimental

### 2.1. Materials

Synthetic graphite (Gr, D90 = 56 µm, TIMREX KS75, <75 µm) was obtained from TIMCAL Graphite and Carbon. A graphitizable binder (GB) consisting of a coal tar pitch with a softening point (SP) of 110°C (Química del Nalón, S.A) was also ground and sieved to <75 µm. Pluronic F127 (Sigma-Aldrich) was used as an additional raw material for the paste formulation.

Metal precursors used for catalyst synthesis included nickel(II) nitrate hexahydrate [Ni(NO_3_)_2_·6 H_2_O, ≥97%], cerium(III) nitrate hexahydrate [Ce(NO_3_)_3_·6 H_2_O, 99%], nickel(II) chloride [NiCl_2_, 98%], iridium(III) chloride monohydrate [IrCl_3_·H_2_O, 99.9%], and platinum(IV) chloride [PtCl_4_, ≥99.9%] provided by Sigma-Aldrich. Cobalt(II) nitrate hexahydrate [Co(NO_3_)_2_·6 H_2_O, ≥98%] was supplied by Honeywell/Fluka, and iron(II) chloride tetrahydrate [FeCl_2_·4 H_2_O, EMSURE] by Supelco. Boric acid [H_3_BO_3_, Panreac, CODEX] and ammonium chloride [NH_4_Cl, ≥99.5%, Sigma-Aldrich] were also used.

Alkaline electrolytes for catalysis (1 M KOH, 1 M KOH +0.5 M NaCl, and 1 M KOH + synthetic seawater) were prepared using KOH (EMSURE), NaCl (Supelco), and synthetic seawater (Aldrich). All solutions were prepared with ultrapure water (Milli-Q).

### 2.2. 3D supports preparation

First, a 25 wt.% aqueous solution of Pluronic F127 was prepared and maintained at 4°C for 12 h to achieve complete dissolution, forming a hydrogel. Subsequently, a solid content of up to 60 wt.%, composed of a Gr-GB mixture in a 47:13 ratio, was incorporated to obtain the printing paste. This formulation follows the same procedure as our previously reported paste [27], but with a higher solid loading (60 wt.% instead of 51 wt.%). The resulting water-based paste was loaded into disposable 3 mL plastic syringes (Adhesive Dispensing) and mounted in a custom-built DIW printer [34]. It was extruded through a 610 µm nozzle tip at 10 mm s^−1^ to produce grid structures (10 × 15 × 2.7 mm) with 50% infill composed of six stacked filament layers with a 26% overlap to ensure good interlayer contact. The as-printed 3D electrodes were dried at 60°C for 24 h and then carbonized in a tubular furnace at 1°C min^−1^ up to 800°C for 1 h under an inert N_2_ atmosphere (200 mL min^−1^), and labelled as 3D-Gr. Larger 3D grids (20 × 25 × 2.7 mm) were also fabricated using the same DIW printing procedure described above for preliminary scalability assessment.

### 2.3. Catalysts preparation

The 3D-Gr all-carbon structures served as support to obtain active catalysts for the OER and HER using routes previously described in the literature.

For the HER catalyst, NiCo-CeO_2_ was electrodeposited onto the 3D-Gr structure using a procedure based on the method reported by Sun et al. [32]. A positive current density of 20 mA cm^−2^ was applied to 3D-Gr for 10 min to induce NO_3_^−^ intercalation (yielding NO_3_^−^/ 3D-Gr as an intermediate sample), followed by a cathodic deposition at −20 mA cm^−2^ for 20 min to deposit NiCo(OH)_x_-CeO_2_. Both electrochemical steps were carried out consecutively in a three-electrode cell with an aqueous solution containing 0.08 M Ni(NO_3_)_2_·6 H_2_O, 0.01 M Co(NO_3_)_2_·6 H_2_O, and 0.01 M Ce(NO_3_)_3_·6 H_2_O as electrolyte. Ag/AgCl (3.5 M KCl) and a graphite rod (Schunk) were used as reference and counter electrodes, respectively, while the 3D-Gr working electrode was clamped in a graphite holder (immersed area ~10 × 10 × 2.7 mm). After the electrochemical deposition, the resulting NiCo(OH)_x_-CeO_2_ / 3D-Gr was rinsed with Milli-Q water and treated at 500°C (5°C min^−1^) for 2 h under an inert atmosphere (95:5 N_2_:H_2_, 200 mL min^−1^) to obtain the final HER catalyst, labelled as NiCo-CeO_2_ / 3D-Gr. To evaluate both the role of CeO_2_ incorporation and the effect of the NO_3_^−^ intercalation step, reference catalysts were prepared following the same electrodeposition and thermal treatment route. Specifically, a NiCo/3D-Gr catalyst was synthesized by excluding Ce(NO_3_)_3_·6 H_2_O from the electrolyte, while an additional NiCo-CeO_2_ / 3D-Gr sample was obtained by omitting the initial intercalation step. For comparison with a conventional substrate, NiCo-CeO_2_ catalyst was also prepared on Toray Carbon Paper (TCP) and is referred to as NiCo-CeO_2_ / TCP.

For the OER catalyst, NiFe was electrodeposited onto the 3D-Gr structure following a route adapted from Ashraf et al. [33]. A negative current density of 40 mA cm^−2^ was applied to the 3D-Gr working electrode for 10 min in a three-electrode cell as described above. In this case, the electrolyte consisted of an aqueous suspension of 0.25 M NiCl_2_, 0.20 M FeCl_2_·4 H_2_O, 0.81 M H_3_BO_3_ (pH buffer), and 0.56 M NH_4_Cl (to increase ionic conductivity) at 60°C. The deposited material was subsequently dried at 60°C for 24 h to yield the final OER catalyst, NiFe/3D-Gr. As in the previous case, the NiFe catalyst was also prepared on TCP, yielding the corresponding NiFe/TCP electrode.

A noble-metal Pt catalyst was loaded onto the 3D-Gr structure to serve as the benchmark HER catalyst. The 3D-Gr sample was immersed for 5 min in a 0.01 M PtCl_4_ solution, dried overnight at 60°C, and then treated at 500°C (5°C min^−1^) for 2 h under an inert atmosphere (95:5 N_2_:H_2_, 200 mL min^−1^) to obtain the reference catalyst, Pt/3D-Gr [35]. For the OER benchmark, an Ir-based catalyst was prepared following an analogous immersion procedure using a 0.01 M IrCl_3_·H_2_O solution. The resulting material was dried and subsequently calcined in air at 300°C for 1 h, yielding the final reference catalyst, IrO_2_ / 3D-Gr [36].

### 2.4. Materials characterization

The morphology and energy-dispersive X-ray spectroscopy (EDS) of the as-prepared samples were examined by scanning electron microscopy (SEM) using a Quanta FEG 650 instrument (FEI Company, United States). X-ray diffraction (XRD) measurements were carried out directly on the 3D structures using a Bruker D8 Advance diffractometer (Bruker, United States) equipped with a Cu K_α_ X-ray source. XPS spectra were recorded on a SPECS system (Germany) operating under a pressure of 10^−7^ Pa with a monochromatic Al K_α_ X-ray source (175 W). Data were recorded at a pass energy of 50 eV for high-resolution scans, binding energies were calibrated to Csp^2^ peak at 284.5 eV, and Shirley baseline correction was applied for all spectra using CasaXPS software. The amount of each metal in the electrodes was determined by inductively coupled plasma mass spectrometry (ICP-MS, Agilent 7700x, Agilent Technologies, United States). The digestion of samples was performed in an advanced microwave digestion system (Ethos 1, Milestone, Italy) in acidic solutions (HCl: HNO_3_:H_2_O, 3: 1: 2). Additionally, ICP-MS analysis was performed on the electrolytes recovered after 200 h stability tests to assess possible metal leaching from the catalysts.

The electrical conductivity of 40 mm-long 3D-Gr filaments, obtained after carbonization, was measured by a 4-point probe method applying a constant current between 2 and 30 mA using a VMP-3e Biologic potentiostat (France). Silver paste was applied to the filament cross-section edges to minimize the contact resistance. Up to ten filaments were measured.

To evaluate the mechanical properties of the all-carbon DIW electrodes, six cylindrical specimens (diameter: 10 mm, height: 10 mm) featuring 1 perimeter and 50% infill were printed using the 3D-Gr formulation and postprocessed similarly to the grid-like electrode geometries. Uniaxial compressive strength was then measured in a universal testing machine (MTS, Sinergy U.S.A. 100N load cell) at 0.05 mm s^−1^.

### 2.5. Electrochemical techniques

All the electrochemical experiments were performed on a VMP-3e Biologic potentiostat. The catalytic activity towards OER and HER was determined using a three-electrode configuration, in which a graphite rod served as the counter electrode and an Ag/AgCl (3.5 M KCl) electrode as the reference. Each as-prepared sample secured in a graphite holder was used as the working electrode. For current density calculations, an effective geometric area of 2 cm^2^ was considered, corresponding to the two faces of the 3D-Gr structure (immersed dimensions ~10 × 10 × 2.7 mm). Electrochemical performance is presented in terms of geometric current density versus potential rather than mass-activity, as the aim of this work is to assess the performance of 3D-Gr based electrodes from a practical application-orientated perspective [37]. The catalytic activity was tested in two different alkaline electrolytes (25 mL), 1 M KOH and 1 M KOH +0.5 M NaCl. Linear sweep voltammetry (LSV, 1 mV s^−1^) and chronopotentiometry (CP) experiments were performed to assess the activity and stability of the catalysts. All potentials applied were calibrated to the reversible hydrogen electrode (RHE) using the equation: E_RHE_ = E_Ag/AgCl_ +0.205 + 0.059 × pH, and 85% iR compensation was applied for all electrochemical measurements. Each measurement was repeated three times, and the curve presented is representative of the obtained data. The overpotentials and Tafel slopes are shown together with their corresponding standard deviations. Tafel slopes are reported only as comparative kinetic descriptors, given the strict conditions required for their meaningful mechanistic interpretation [38,39]. Double-layer capacitance (C_dl_) was estimated from cyclic voltammetry in a non-faradaic potential region at different scan rates ranging from 1 to 50 mV s^−1^. Due to the lack of reliable specific capacitance values (C_s_) for these heterogeneous 3D electrodes, ECSA could not be robustly determined from C_dl_ and is therefore not reported [40]. Electrochemical impedance spectroscopy (EIS) measurements were also performed at frequencies of 100 kHz to 10 mHz with an amplitude of 10 mV. EIS spectra were acquired at potentials where HER and OER occur to evaluate the charge transfer resistance (R_ct_).

The performance and stability of the supported catalysts were evaluated in overall water splitting using a custom-made two-electrode cell, with the NiFe/3D-Gr and 3D-NiCo-CeO_2_/3D-Gr acting as anode and cathode, respectively. To better approximate realistic operating conditions, 25 mL electrolyte consisting of 1 M KOH in synthetic seawater was also employed. During its preparation, white precipitates, mainly Ca(OH)_2_ and Mg(OH)_2_, were formed and removed prior to use.

## 3. Results and discussion

**Figure 1.**
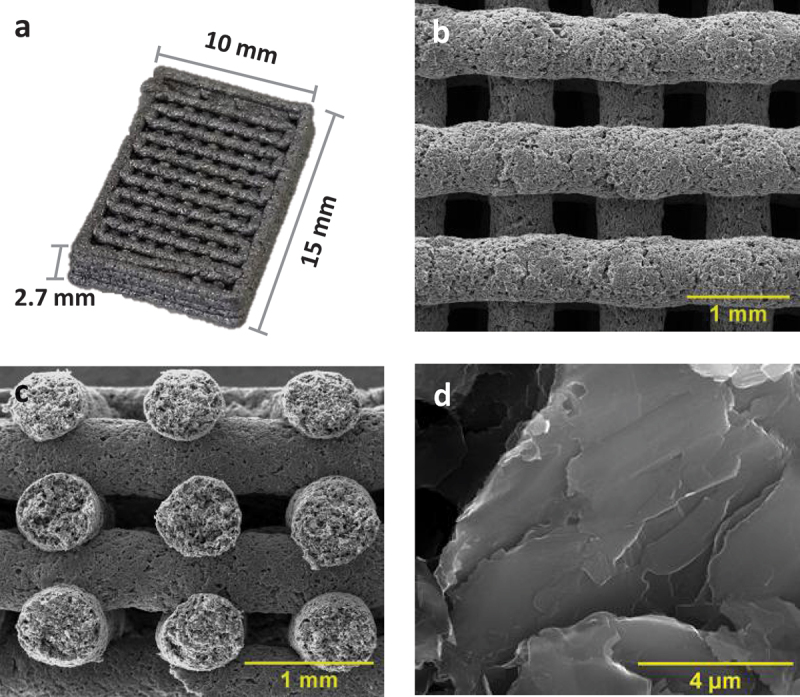
Macroscopic aspect of 3D-Gr (a), and SEM images showing grid-like structure (b), the corresponding cross-section of the filaments (c), and a higher-magnification image revealing the stacked, flake-like graphite particles (d).

The 3D-structured all-carbon DIW electrodes used as support for OER and HER electrocatalysts were obtained by processing a high-solids-loading (60 wt.%) water-based paste composed primarily of graphite and graphitizable binder powders. After DIW and the post-processing steps, a grid-shaped structure with negligible shrinkage is obtained, resulting in final dimensions (10 × 15 × 2.7 mm) very close to those of the as-printed electrode (Figure 1a). The major composition of these DIW structures is graphite consolidated by approximately a 10 wt.% of carbon derived from the graphitizable binder [27]. The filaments of these grid-like all-carbon DIW structures exhibit an outstanding electrical conductivity of 10,700 S m^−1^, which is a 26% higher than that obtained from the previously reported lower-solids-loading paste (51 wt.%) [27] (Figure S1). This value of electrical conductivity, arising from the graphitic nature of the final electrode, is by far larger than carbon-loaded FDM filaments or other AM technologies [30,31], thus showing an excellent opportunity to use them as catalyst supports. In addition, the uniaxial compressive strength reaches 5 MPa, representing a 30% increase compared with the lower-solids-loading one [27], which demonstrates the huge potential for optimization by tuning composition (Figure S1). SEM images of the 3D-Gr support showed successive layers of carbon filaments with a diameter of 615 ± 22 µm, arranged with alternating 90° orientation and an inter-filament spacing of 456 ± 15 µm (Figure 1b,c). Higher-magnification images further revealed that the filaments are composed of stacked, flake-like graphite particles, which are preferentially oriented along the printing direction (Figure 1d). The selection of this simple grid-like geometry and relatively thick nozzle size represents a compromise between an increased surface-to-volume ratio and mechanical robustness. It is beyond of the scope of this preliminary work increase the complexity of the structure by varying nozzle size, inter-filament spacing or filament trajectories as the main goal here is to test them as catalysts support.

XRD analysis of the 3D-Gr showed its graphitic structure, displaying a diffraction pattern consistent with graphite powder (COD 9,011,577, space group P/63 m m c) [41,42] (Figure S2). Complementary ICP-MS measurements detected non-relevant concentrations (<0.05 ppm) of Ni, Fe, Co, and Ce, indicating the absence of significant metallic impurities in the support before electrodeposition. This ensures that the transition metals detected in subsequent analyses can be attributed exclusively to the electrodeposition process. To evaluate the electrocatalytic activity of 3D-Gr, linear polarization curves were recorded in the potential regions of HER and OER in 1 M KOH (Figure S3). No significant current densities were achieved, indicating that the structure exhibits an inert behavior toward water splitting reactions. Taken together with the excellent mechanical stability and electrical conductivity previously reported for these 3D materials [27], these results support considering 3D-Gr as a potential platform for catalyst preparation. Moreover, this extrusion-based fabrication approach inherently allows a wide range of design flexibility, providing opportunities for future optimization, such as incorporating various functional materials, including carbon-based structures (e.g. graphene, carbon nanotubes, carbon black) or catalyst precursors, into the filaments during the 3D processing.

The preparation of the NiCo-CeO_2_/3D-Gr HER catalyst involved a first electrochemical step consisting of nitrate intercalation, previously reported to enhance the contact between catalyst and support when using dense graphite plates [32,43]. To assess whether this intercalation effect also influences the 3D-Gr structure, the sample was compared before and after intercalation. While XRD of the 3D-Gr support after nitrate intercalation, labelled as NO_3_-/3D-Gr, only showed subtle changes in the width of the main graphite peak at 26.5° (Figure S4a), Raman spectroscopy provided clearer evidence of this structural modification, as the D band intensity increased significantly, with the I_D_/I_G_ ratio rising from 0.04 in pristine 3D-Gr to 0.20 for NO_3_^−^/3D-Gr (Figure S4b). Electrochemical characterization of the support by cyclic voltammetry (CV) also evidenced the effect of nitrate intercalation, showing a significant increase in capacitive current for NO_3_^−^/3D-Gr compared to 3D-Gr, indicative of a larger active surface area (Figure S4c). All these results suggest that nitrate intercalation occurs in the 3D electrodes, which are subsequently employed as working electrodes for the electrodeposition of NiCo-CeO_2_.

**Figure 2.**
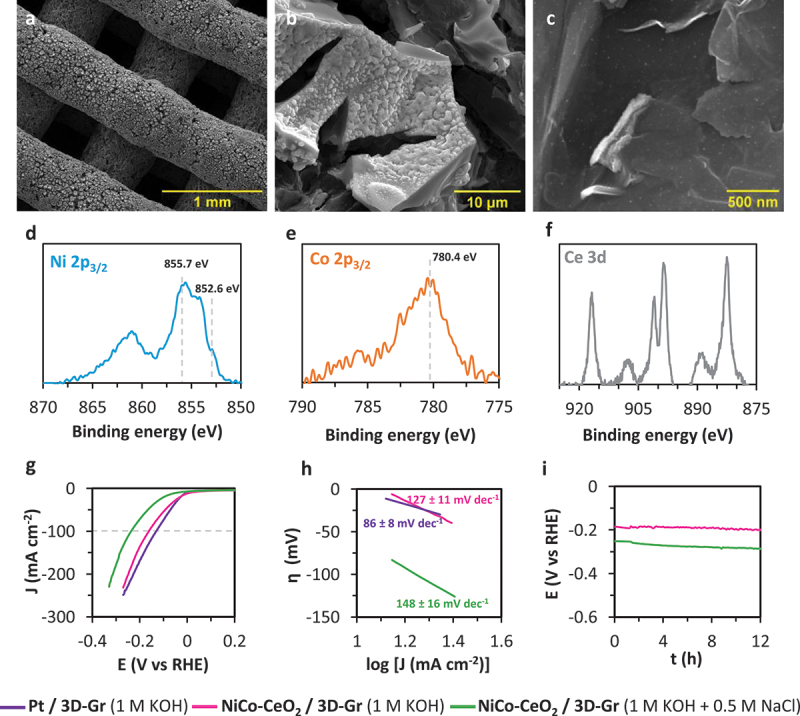
SEM images (a-c) and high-resolution XPS spectra (d-f) of the NiCo-CeO_2_ / 3D-Gr sample. iR-corrected polarization curves recorded at 1 mV s^−1^ (g) with their corresponding Tafel plots (h). Chronopotentiometric measurements of NiCo-CeO_2_ / 3D-Gr performed at −100 mA cm^−2^ for 12 h in both electrolytes (i).

After cathodic electrodeposition of metals (Ni, Co and Ce) followed by thermal treatment, SEM images of NiCo-CeO_2_ / 3D-Gr revealed that the carbon filaments were well coated with a granular deposit, denser on the outermost filaments but also present in underlying ones (Figure 2a). Large agglomerates were observed on the filaments (Figure 2b), coexisting with a finer dispersion of metal-derived nanoparticles in areas where the carbon surface remained exposed, shown in Figure 2c. EDX mapping confirmed that Ni, Co, and Ce are homogenously distributed throughout the 3D-Gr surface (Figure S5). Analysis of the total metal content by ICP-MS indicated concentrations of 3.37 wt.% Ni, 0.47 wt.% Co, and 1.06 wt.% Ce relative to the entire electrode (~160 mg) (Table S1, including standard deviations). Notably, as deposition is concentrated on the outermost layers, these values underestimate the real metal content at the filament surface. A more meaningful metric is the atomic ratio of the metals, Ni:Co:Ce = 8:2:1, reflecting the preferential deposition of Co over Ce despite equal precursor concentrations. In the case of the Pt/3D-Gr electrode used as the HER benchmark, ICP-MS gave a Pt content of 0.27 wt.%, consistent with the low Pt loadings targeted for practical HER electrodes [44].

XRD of NiCo-CeO_2_ / 3D-Gr showed the characteristic graphite pattern, along with peaks at 51.8° and 76.4°, which can be primarily assigned to metallic Ni (COD 9,008,476, space group F m-3 m) (Figure S6). However, due to peak broadening and the very similar diffraction peak positions of metallic Co (COD 9,008,466, space group F m-3 m), the presence of metallic Co or a NiCo alloy, as previously reported for similar systems [32], cannot be excluded. The diffraction peak at 28.5° corresponds to CeO_2_ (COD 9,009,008, space group F m −3 m) [45] and represents its more intense plane (111). Additional Ni, Co and CeO_2_ peaks were not clearly identified, likely due to the low metal content relative to the dominant crystalline graphite support. Complementary XPS analysis, with the survey spectra shown in Figure S7, indicated that Ni, Co, and Ce were predominantly present on the electrode surface as oxidized species. High-resolution spectra of Ni and Co were analyzed based on the positions of their main binding energy peaks, due to the inherent complexity arising from multiplet splitting and overlapping contributions in 3d transition metal spectra [46]. The high-resolution Ni 2p_3/2_ spectrum (Figure 2d) exhibits a main peak at ~855.7 eV, consistent with Ni^2+^ in Ni(OH)_2_, together with a minor shoulder at ~852.6 eV, suggesting a small contribution from metallic Ni [47]. The Co 2p spectrum (Figure 2e) also shows features characteristic of Co^2+^ species, with maximum position centered at 780.4 eV, likely corresponding to Co(OH)_2_ [48]. In the case of Ce, Figure 2f displays the typical six-component spectrum of CeO_2_, including its distinctive feature at ~917 eV [49].

The HER performance of NiCo-CeO_2_/3D-Gr was evaluated in 1 M KOH by linear sweep voltammetry. The catalyst exhibits high activity, reaching a current density of −100 mA cm^−2^ at an overpotential of 155 ± 13 mV, comparable to that for the Pt/3D-Gr of 135 ± 14 mV (Figure 2g). Control experiments, shown in Figure S8, demonstrated that the electrodes prepared either without the nitrate intercalation step or without CeO_2_ (NiCo/3D-Gr) exhibit higher HER overpotentials (see values in Table 1), indicating poorer catalytic activity. The positive effect of nitrate intercalation is consistent with previous reports [32,43], while the enhanced activity upon CeO_2_ incorporation has been attributed to accelerated water dissociation [32,43]. For comparison purposes, the NiCo-CeO_2_ catalyst was also prepared on Toray carbon paper. As shown in Figure S8 and summarized in Table 1, NiCo-CeO_2_ / TCP exhibits a HER overpotential of 341 mV, higher than that of NiCo-CeO_2_ / 3D-Gr, indicating that this all-carbon 3D electrode can be effectively used as catalyst support. This interesting difference in HER performance between NiCo-CeO_2_ / TCP and NiCo-CeO_2_ / 3D-Gr may reflect support-related effects, potentially including the conductivity of the 3D graphitic structure and its influence on catalyst deposition and electrode operation, enhanced exposure of active sites enabled by the grid-like geometry, and/or improved bubble release. However, clarifying the relative contribution of these and other factors will require dedicated future work. In addition, the electrochemical properties of the NiCo-CeO_2_ / 3D-Gr were also analyzed by double-layer capacitance (C_dl_) and electrochemical impedance spectroscopy (EIS) (Figure S9). An increase in the C_dl_ value was observed after NiCo-CeO_2_ deposition compared to the bare 3D- Gr support (from 54 mF cm^−2^ to 155 mF cm^−2^). This trend is consistent with previous reports in which CeO_2_ incorporation has been associated with increased C_dl_ values [64]. Furthermore, when measured at HER operating potentials (−0.15 V), EIS reveals a closed semicircle for NiCo-CeO_2_ / 3D-Gr sample, consistent with high charge-transfer kinetics in the presence of the catalyst.

**Table 1.** Comparison of overpotentials for different HER and OER catalysts in alkaline and alkaline – saline electrolytes.

Material	3DP technique	Electrolyte	Overpotential @ 100 mA cm^−2^	Ref
**HER**				
**Catalyst**				
NiCo-CeO_2_ / 3D-Gr	DIW	1 M KOH	155 mV	This work
NiCo-CeO_2_ / 3D-Gr without NO_3_^−^	DIW	1 M KOH	274 mV	
NiCo / 3D-Gr	DIW	1 M KOH	275 mV	
NiCo-CeO_2_ / TCP	–	1 M KOH	341 mV	
NiCo-CeO_2_ / 3D-Gr	DIW	1 M KOH +0.5 M NaCl	221 mV	
NiCo / NM	–	1 M KOH	236 mV	[50]
Ni@Cu / 3D PyC	DLP	1 M KOH	264 mV	[51]
3DC-Fe-CNTs-Ni/Ni(OH)_2_	DIW	1 M KOH	164 mV	[52]
3DP NiMo_6Layers	DIW	1 M KOH	227 mV	[53]
Cu_3_P-FeP@CC	–	1 M KOH +0.5 M NaCl	260 mV	[54]
P-Ni_4_Mo	–	1 M KOH +0.5 M NaCl	250 mV	[55]
Ni_3_Se_2_@MoO_3_/CF	–	1 M KOH + seawater	242 mV	[56]
**OER**				
NiFe / 3D-Gr	DIW	1 M KOH	318 mV	This work
NiFe / TCP	–	1 M KOH	402 mV	
NiFe / 3D-Gr	DIW	1 M KOH +0.5 M NaCl	309 mV	
CESS	SLM	1 M KOH	332 mV	[57]
3DP-Ni-600°C_1h	DIW	1 M KOH	495 mV	[58]
NiCo / NM	–	1 M KOH	372 mV	[50]
NiCo_2_S_4_/3DP	DIW	1 M KOH	277 mV	[59]
3DP GC / NiFeP-24 L	DIW	1 M KOH	340 mV	[60]
NiMoN @ NiFeN	–	1 M KOH	277 mV	[61]
NiMoN @ NiFeN	–	1 M KOH +0.5 M NaCl	286 mV	[61]
NiIr-LDH	–	1 M KOH +0.5 M NaCl	286 mV	[62]
FeOOH @ Ni(OH)_2_	–	1 M KOH + seawater	325 mV	[63]

To assess the effect of chloride ions, experiments were also conducted in a chloride-containing electrolyte (1 M KOH +0.5 M NaCl, approximating the chloride concentration of seawater). In this electrolyte, a slight decrease in performance was observed, with a higher overpotential and a reduction in the maximum current density. Nevertheless, NiCo-CeO_2_ / 3D-Gr still achieved −100 mA cm^−2^ at a relatively low overpotential of 221 ± 29 mV and exhibited a Tafel slope similar to that observed in 1 M KOH electrolyte (Figure 2h). The overpotential values obtained in this work are comparable to those reported in the literature, including materials deposited on Ni mesh [50], other 3D carbon-based electrodes fabricated via DPL [51] or DIW [52], as well as other catalysts tested in alkaline – saline electrolytes [54–56], as shown in Table 1. Stability tests were conducted in both electrolytes at a current density of −100 mA cm^−2^ (Figure 2i). The potential remained essentially stable for 12 h, with a decay of only 15 mV in 1 M KOH and 28 mV in 1 M KOH +0.5 M NaCl, demonstrating excellent durability of the catalysts even at a relatively high current density, well above the −10 mA cm^−2^ widely used for stability tests in lab-scale [65–67].

**Figure 3.**
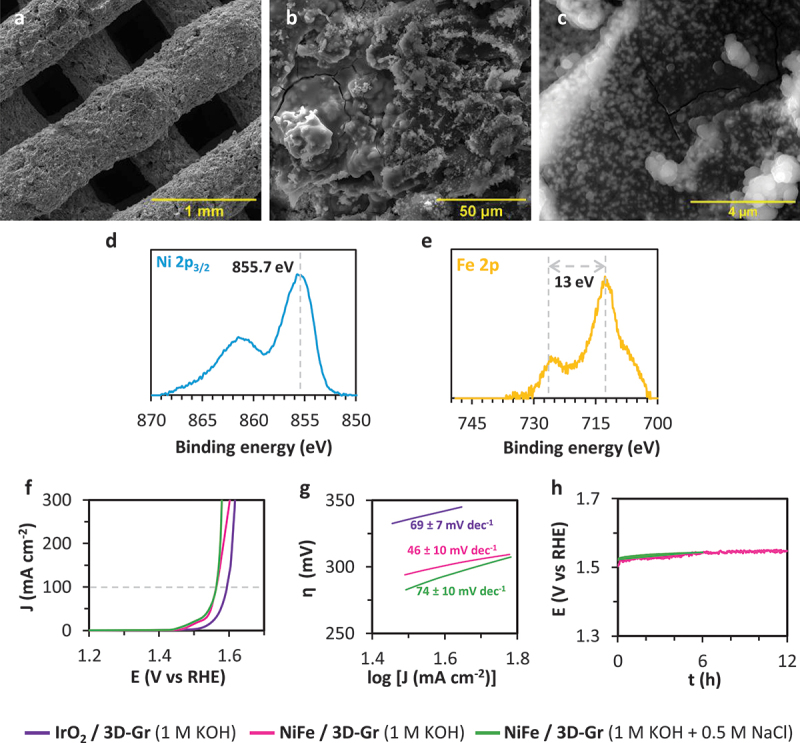
SEM images (a-c) and high-resolution XPS spectra (d,e) of the NiFe / 3D-Gr sample. iR-corrected polarization curves recorded at 1 mV s^−1^ (f) with their corresponding Tafel plots (g). Chronopotentiometric measurements of NiFe / 3D-Gr performed at 100 mA cm^−2^ for 12 h in both electrolytes (h).

On the other hand, the NiFe/3D-Gr OER catalyst was obtained through a simple one-step electrodeposition. As previously observed for the NiCo-CeO_2_ / 3D-Gr system, the deposited metals were mainly concentrated on the outer filaments, while still extending to the underlying ones (Figure 3a). At higher magnification, the coating was revealed as a thin layer composed of finely dispersed nanoparticles (Figure 3b,c). EDX mapping further confirmed the homogeneous distribution of Ni and Fe throughout the sample (Figure S10). Quantification of the metal loading by ICP-MS yielded 5.85 wt.% Ni and 1.72 wt.% Fe with respect to the total electrode mass (~160 mg) (Table S1). From these values, an atomic Ni:Fe ratio of 3:1 was obtained, indicating a preferential deposition of Ni during the electrodeposition process. The IrO_2_ / 3D-Gr benchmark contained 0.10 wt.% Ir, consistent with the low loadings pursued for practical Ir-based electrodes [68].

XRD analysis of NiFe/3D-Gr showed a new reflection at 75.4°, absent in the pristine 3D-Gr pattern, which can be attributed to the overlapping contributions of metallic Ni (COD 9,012,968, space group F m −3 m) and Fe (COD 9,015,937, space group F m −3 m) (Figure S11) [69]. Additional reflections expected for these metals at 44.4° and 54.6° fall within regions where graphite already exhibits intense peaks, making them difficult to resolve. A slight broadening of these graphite peaks could, however, suggest the presence of these metallic phases. Despite the detection of metallic species by XRD, XPS analysis of the NiFe/3D-Gr surface (survey spectra in Figure S12) revealed the presence of oxidized Ni and Fe species. In the high-resolution Ni spectrum (Figure 3d), the maximum binding energy at 855.7 eV is consistent with the presence of Ni(OH)_2_ [47], while the Fe spectrum shows two main peaks separated by 13 eV (Figure 3e), in agreement with the expected for Fe^3+^ species [70,71].

The OER activity of NiFe/3D-Gr was evaluated in both 1 M KOH and 1 M KOH +0.5 M NaCl electrolytes and compared to the reference IrO_2_ / 3D-Gr. While IrO_2_ is not the most active OER catalyst in alkaline media, it is commonly used as a reference for comparison. Under the experimental conditions, IrO_2_ / 3D-Gr exhibited an overpotential of 371 ± 11 mV to achieve 100 mA cm^−2^, whereas NiFe/3D-Gr reached the same current density at a significantly lower overpotential of 318 ± 10 mV (Figure 3f). Following the electrochemical characterization in 1 M KOH, and similarly to the NiCo-CeO_2_ system, NiFe was also prepared on TCP for comparison with a conventional support. The NiFe/TCP electrode exhibits a higher overpotential and a larger Tafel slope than NiFe/3D-Gr (Figure S13, Table 1), indicating poorer OER activity and further supporting the suitability of the DIWed electrode as a catalyst support. As observed for HER, this difference may also reflect support-related effects, whose contribution to the overall OER performance merits further investigation in future work. The estimation of the C_dl_ shows that a high capacitance value (66 mF cm^−2^) is maintained after NiFe incorporation on the 3D-Gr support (Figure S14a-c). In addition, EIS analysis reveals that NiFe/3D-Gr displays a small semicircular feature at OER potential (1.57 V), consistent with fast charge-transfer in the presence of the catalyst (Figure S14d).

Notably, NiFe/3D-Gr maintained a comparable performance (309 ± 17 mV @ 100 mA cm^−2^) and Tafel slope values (Figure 3g) in the saline electrolyte, demonstrating robustness under harsh conditions. Importantly, the competing chlorine evolution reaction (ClER) is not expected under these potentials, as it generally occurs at overpotentials of ~480 mV [72], well above the 300–320 mV range observed for the NiFe/3D-Gr catalyst. These overpotentials are highly competitive compared to other catalysts reported in the literature (Table 1), such as 3DP GC/NiFeP-24 L (340 mV at 100 mA cm^−2^) prepared by DIW using graphene and carbon nanotubes [60], or NiMoN @ NiFeN (286 mV at 100 mA cm^−2^) deposited on Ni foam [61]. Stability tests conducted over 12 h at 100 mA cm^−2^ showed a potential increase of only 39 mV in 1 M KOH and 29 mV in 1 M KOH +0.5 M NaCl, confirming the excellent durability of NiFe/3D-Gr under high current densities even in aggressive saline conditions (Figure 3h). Notably, even after this slight potential increase, the overpotential remains below 480 mV, further indicating that ClER is still not expected to occur.

**Figure 4.**
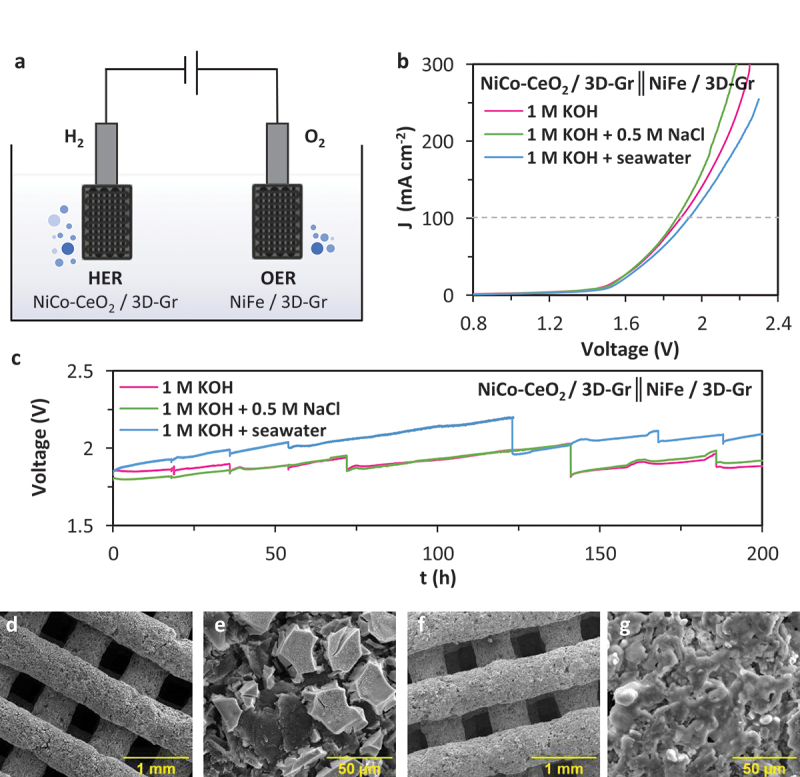
Overall water splitting experiments. A schematic representation of the two-electrode cell (a); iR-corrected polarization curves of the NiCo-CeO_2_/3D-Gr ║NiFe/3D-Gr system (b), long-term stability test by chronopotentiometry at 100 mA cm^−2^ over 200 h (c); and post-catalysis SEM images of NiCo-CeO_2_/3D-Gr (d,e) and NiFe/3D-Gr (f,g) electrodes.

To further demonstrate the practical applicability of the catalyst/3D-Gr systems, overall water and seawater splitting was evaluated in a two-electrode configuration, schematically illustrated in Figure 4a, with NiCo-CeO_2_ / 3D-Gr and NiFe/3D-Gr serving as cathode and anode, respectively. To drive increasing current densities, NiCo-CeO_2_ / 3D-Gr ║NiFe/3D-Gr system required 1.71 V, 1.88 V, and 2.18 V to reach 50, 100, and 250 mA cm^−2^, respectively, in 1 M KOH (Figure 4b). Similar results were obtained when the electrolyte consisted of 1 M KOH +0.5 M NaCl, indicating that the electrode activity is not compromised by the presence of chlorides, one of the main challenges in seawater splitting.

Moreover, to better reproduce real seawater electrolysis conditions, 1 M KOH in synthetic seawater was used as electrolyte. In this case, the cell voltage required to reach 100 mA cm^−2^ increased to 1.93 V (Figure 4b), which is still considered a good performance result [73–75]. To gain further insight into the origin of the increased cell voltage, additional HER and OER measurements were performed in a three-electrode configuration using the same electrolyte (Figure S15). From this analysis, it can be deduced that the increase in the overall cell voltage arises mainly from a slight increase in the OER overpotential (349 mV at 100 mA cm^−2^, compared to 309 mV in 1 M KOH +0.5 M NaCl at the same current density), as no significant changes in the HER overpotentials are observed in the two saline electrolytes (222 and 221 mV at 100 mA cm^−2^). This difference in OER overpotentials could be attributed to the higher ionic complexity of the synthetic seawater electrolyte.

At this point, it is worth recalling that all potentials are corrected for ohmic drop. Figure S16 displays the polarization curves of the two-electrode systems without iR compensation, showing higher cell voltages. Importantly, this increase does not indicate that the electrodes operate at intrinsically higher anodic or cathodic potentials; rather, it reflects the additional voltage required to overcome the ohmic resistance of the cell [76]. In our case, the approximately 400 mV difference corresponds to an ohmic resistance of about 2 Ω. Although these uncorrected values are rarely included in the literature [77], this work proposes that reporting them is useful for assessing realistic operation, which should be considered in future applied studies. These non-corrected values therefore provide an indication of the practical energy input required when considering system resistance (electrochemical setup-dependent), which becomes increasingly relevant when moving toward large-scale and prototype electrolysis devices [78].

Long-term stability tests were carried out in the three electrolytes (Figure 4c), showing nearly constant cell voltage over 200 h at 100 mA cm^−2^, indicating excellent electrode durability. Minor visible voltage drops correspond to points where electrolyte volume was added to compensate for evaporation, which had caused part of the electrode surface to become partially non-immersed. Moreover, Figure 4d–g show SEM images of the post-catalysis electrodes tested in 1 M KOH +0.5 M NaCl, which show that the carbon filaments remain intact and the deposited metal species are still present, confirming the structural and compositional stability of the electrodes. Additional SEM images of electrodes tested in the other electrolytes are provided in Figure S17 showing a similar structural stability. ICP analysis of the electrolytes, fresh and after 200 h of operation, revealed no increase in the metal concentrations (Ni, Fe, Co, or Ce, Table S2), confirming the outstanding robustness of the electrodes. Only the post-catalysis electrolyte corresponding to the 1 M KOH containing seawater showed slightly higher metal concentrations, which can be attributed to the higher voltages reached in this electrolyte, possibly inducing minor surface dissolution. Nevertheless, less than 1% of each metal content was leached into the electrolyte, demonstrating excellent chemical stability and strong adhesion of the metallic catalysts to the carbon support, even under prolonged electrolysis and in complex electrolyte environments. As a complementary post-catalysis characterization, XPS analysis was focused on the Ni 2p_3/2_ region, as nickel is the predominant metallic element in both catalysts. Nearly identical spectra were obtained after chronopotentiometry experiments in the three electrolytes, all consistent with Ni(OH)_2_ (~855.7 eV). In NiFe/3D-Gr, no changes in Ni 2p_3/2_ peak positions were observed because nickel was already present as Ni(OH)_2_, whereas in NiCo-CeO_2_ / 3D-Gr, slight oxidation occurred due to the initial presence of NiO and metallic Ni (Figure S18). The similarity of the post-catalysis spectra across the different electrolytes confirms that chloride did not induce additional surface modifications, indicating good chemical stability under saline conditions. While a detailed mechanism study of chloride-induced degradation and ClER is beyond the scope of this work, the mentioned electrochemical and chemical stability results underscore the robustness of the 3D-Gr supported catalysts developed and the potential of these 3D carbon supports for developing advanced electrocatalysts. Importantly, the performance reported here was achieved without additional optimization, using a unique support geometry and previously reported electrocatalyst synthesis methods, revealing a significant margin for improvement that opens the door to new studies.

Given the favorable water-splitting activity of the electrodes and their stable operation over 200 h, preliminary scale-up experiments were conducted in a two-electrode configuration using larger 3D grids (20 × 25 × 2.7 mm, Figure S19a). These electrodes exhibited similar current-density voltage response when normalized by geometric area, indicating that the 3D electrodes can be successfully scaled up within the dimensions investigated (Figure S19b).

## 4. Conclusions

These experiments provide a clear proof-of-concept, demonstrating not only that previously reported synthetic strategies can be successfully implemented on 3D-printed graphite supports, but also that these electrodes are suitable for practical two-electrode water splitting configurations. For the HER, NiCo-CeO_2_/3D-Gr exhibited a low overpotential of 155 mV at 100 mA cm^−2^ in 1 M KOH, while the OER catalyst NiFe/3D-Gr reached the same current density at an overpotential of approximately 300 mV in both alkaline and alkaline-saline electrolytes. The comparable activity observed under both electrolyte conditions further confirms the robustness of the catalysts and their tolerance to saline environments. Moreover, outstanding performance and electrode stability were maintained for 200 h of overall water/seawater.

## 5. Future perspectives

DIW opens up opportunities to develop novel 3D structured electrodes for electrochemical applications by increasing the availability of materials with outstanding properties, while keeping low cost and printing simplicity. The graphite-based DIWed electrodes developed here enabled robust and versatile platforms for integrating active materials into functional devices, and also open the door to future studies focused on (i) testing these electrodes under flow conditions to evaluate the advantages of their 3D structure, (ii) exploring variations in the electrode and catalyst composition, and (iii) modifying electrode geometry design.

## Supplementary Material

Supplemental Material
