# Supplementary material for: All-carbon 3D-structured electrodes by direct ink writing as electrocatalyst supports for alkaline freshwater and seawater electrolysis
Source: Sci Technol Adv Mater. 2026 Jun 23;27(1):2688057. doi: 10.1080/14686996.2026.2688057 (PMC13348212; doi:10.1080/14686996.2026.2688057)
Supplement: Supplemental Material [file TSTA_A_2688057_SM2323.docx]

**SUPPLEMENTARY INFORMATION**

**All-carbon 3D-Structured Electrodes by Direct Ink Writing as Electrocatalyst Supports for Alkaline Freshwater and Seawater Electrolysis**

**Lucía Muñiz Muñoz, María González-Ingelmo*, Pablo Rodríguez-Lagar, Miriam López García, Jonathan Ruiz Esquius, Daniel Barreda, Ricardo Santamaría, Clara Blanco, Victoria G. Rocha**

*Instituto de Ciencia y Tecnología del Carbono, INCAR-CSIC, C/Francisco Pintado Fe, 26, Oviedo, 33011, Spain*

**corresponding author: maria.ingelmo@incar.csic.es*





**Figure S1**. Electrical conductivity (grey bars) and uniaxial compressive strength (red bars) of 3D all-carbon electrodes with different solid contents. Data for the 51 wt.% 3D-Gr (40:11) formulation reproduced from [1].

**Figure S2**. XRD profile of the 3D‑Gr structure.


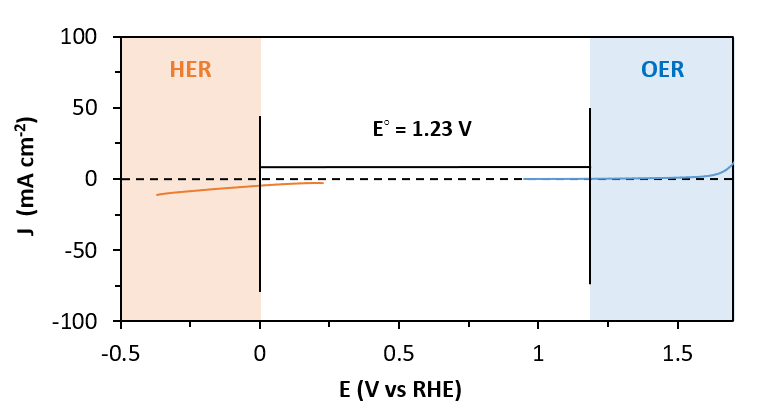


**Figure S3**. 3D-Gr linear polarization curves (1 mV s^-1^) recorded in the potential regions of HER and OER using 1 M KOH as electrolyte.

**Figure S4**. Characterization of the NO_3_^-^ / 3D‑Gr including (a) XRD pattern, (b) Raman spectrum, and (c) CV experiments. 3D‑Gr characterization is also shown for comparison.

**Figure S5**. EDX mapping of NiCo‑CeO_2_ / 3D‑Gr corresponding to carbon, oxygen, cerium, nickel, and cobalt.

**Table S1**. Metal concentrations in the samples determined by ICP-MS. Values are reported together with their standard deviations.

| **Sample** | **Concentration wt. %** | | | | | | |
| --- | --- | --- | --- | --- | --- | --- | --- |
|  | **Ni** | | **Co** | | **Ce** | | **Fe** |
| **NiCo-CeO_2_ / 3D-Gr** | 3.37 ± 0.27 | 0.47 ± 0.04 | | 1.06 ± 0.13 | | - | |
| **NiFe / 3D-Gr** | 5.85 ± 0.26 | - | | - | | 1.72 ± 0.11 | |

**Figure S6**. Comparison of XRD profiles of 3D‑Gr and NiCo-CeO_2_ / 3D‑Gr.

**Figure S7**. XPS survey spectrum of NiCo-CeO_2_ / 3D‑Gr.


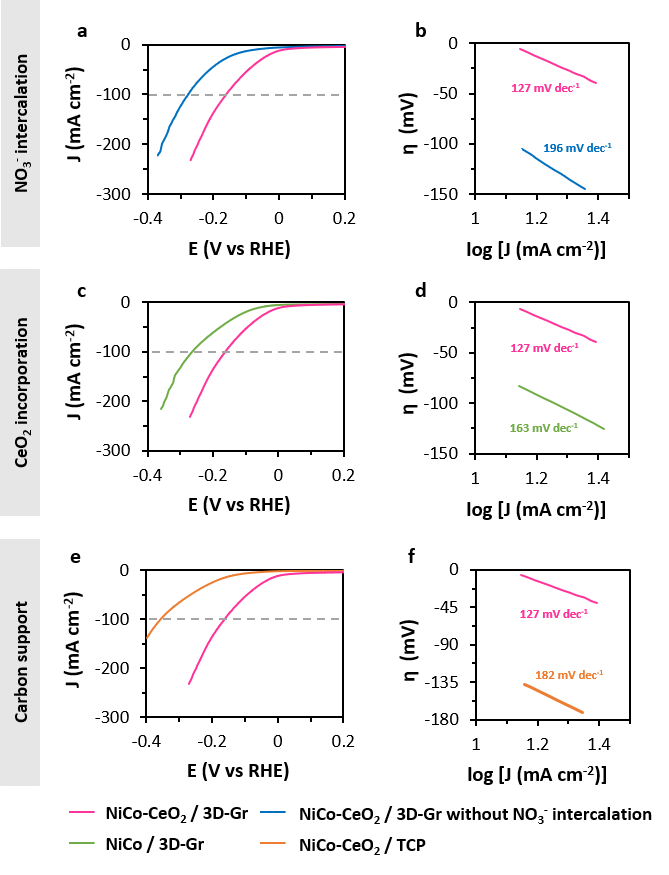


**Figure S8**. Linear polarization curves at 1 mV s^-1^ of the control experiments for the HER activity (a, c, e) with their corresponding Tafel slopes (b, d, f) in KOH 1 M.

**
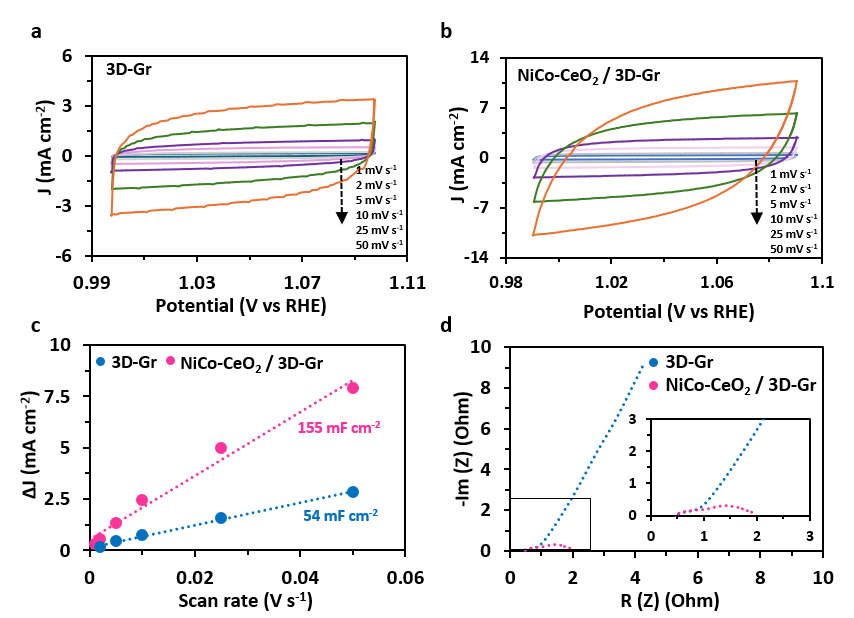
**

**Figure S9.** Cyclic voltammograms collected at scan rates ranging from 1 to 50 mV s^-1^ for 3D‑Gr (a) and NiCo-CeO_2_ / 3D‑Gr (b), together with the corresponding C_dl_ determination (c). Nyquist plots of the samples recorded at a potential of -0.15 V vs RHE (d).

**Figure S10**. EDX mapping of NiFe/ 3D‑Gr corresponding to carbon, oxygen, nickel, and iron.

**Figure S11**. Comparison of XRD profiles of 3D‑Gr and NiFe / 3D‑Gr.

**Figure S12**. XPS survey spectrum of NiFe / 3D‑Gr.


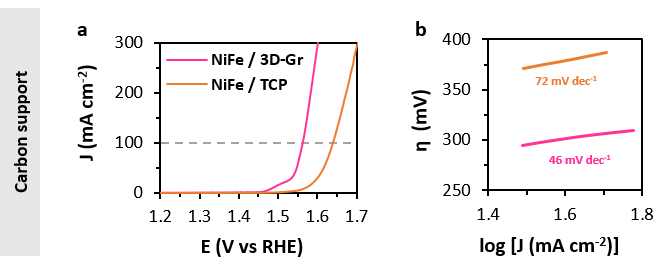


**Figure S13**. LSV comparison of NiFe / TCP and NiFe / 3D-Gr recorded at 1 mV s^-1^ in KOH 1 M (a) with their corresponding Tafel plots (b).


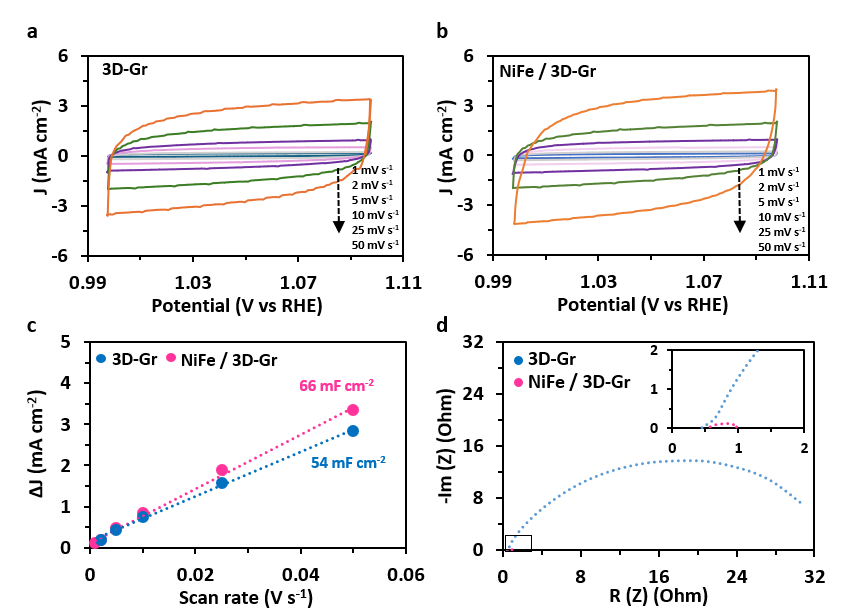


**Figure S14.** Cyclic voltammograms collected at scan rates ranging from 1 to 50 mV s^-1^ for 3D Gr (a) and NiFe / 3D Gr (b), together with the corresponding C_dl_ determination (c). Nyquist plots of the samples recorded at a potential of 1.57 V vs RHE (d).

**Figure S15**. iR-corrected polarization curves recorded at 1 mV s^-1^ for NiCo-CeO_2_ / 3D‑Gr (a) and NiFe / 3D‑Gr (c) with their corresponding Tafel plots (b,d) in 1 M KOH + seawater.

**Figure S16**. Overall seawater splitting performance of the NiCo-CeO_2_ / 3D‑Gr ║NiFe / 3D‑Gr system with and without iR compensation.


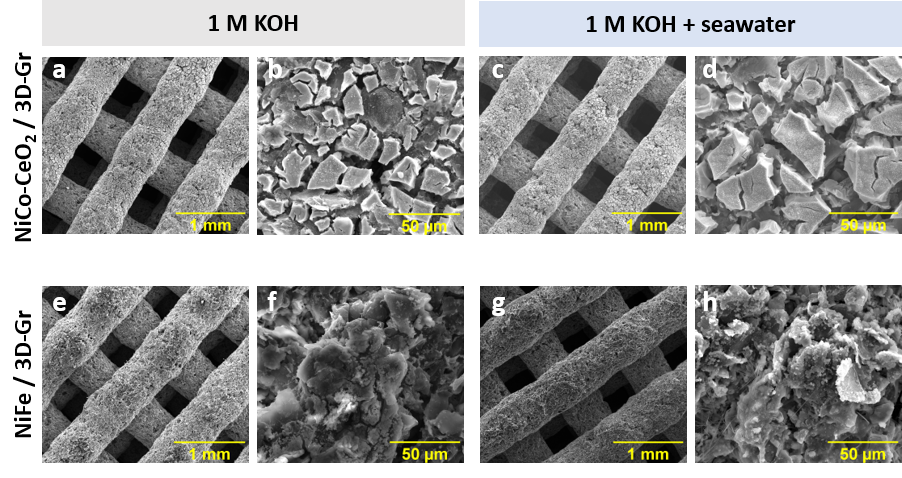


**Figure S17**. Post-catalysis SEM images of NiCo-CeO_2_ / 3D‑Gr (a-d) and NiFe / 3D‑Gr (e-h) electrodes after 200 h at 100 mA cm^-2^ in 1 M KOH and 1 M KOH + seawater.

**Table S2**. Metal concentrations detected in the electrolytes after 200 h of overall water splitting, determined by ICP-MS.

| **Electrolyte** | | **Concentration (ppm)** | | | | |
| --- | --- | --- | --- | --- | --- | --- |
|  |  | **Ni** | **Co** | **Ce** | | **Fe** |
| **1 M KOH** | **Fresh** | 0.14 ± 0.00 | 0.02 ± 0.00 | | 1.08 ± 0.00 | 0.14 ± 0.01 |
|  | **Post-catalysis** | 0.65 ± 0.05 | 0.07 ± 0.01 | | 1.36 ± 0.01 | 0.49 ± 0.01 |
| **1 M KOH +**  **0.5 M NaCl** | **Fresh** | 0.27 ± 0.09 | 0.02 ± 0.00 | | 1.10 ± 0.00 | 0.62 ± 0.07 |
|  | **Post-catalysis** | 0.51 ± 0.00 | 0.09 ± 0.01 | | 1.21 ± 0.02 | 0.44 ± 0.01 |
| **1 M KOH + seawater** | **Fresh** | 0.35 ± 0.05 | 0.02 ± 0.00 | | 1.19 ± 0.02 | 0.35 ± 0.07 |
|  | **Post-catalysis** | 6.13 ± 0.59 | 0.78 ± 0.08 | | 4.29 ± 0.21 | 1.54 ± 0.03 |

The percentage of leached metal was determined using the following equation:

$$\text{\% }Leached\text{ }metal\text{=}\frac{(C_{metal,\text{ }post\text{-}catalysis}\text{-}C_{metal,fresh})\times V_{electrolyte}}{m_{metal,\text{ }catalyst}}\times100$$

Where C_metal,fresh_ and C_metal,post-catalysis_ are the metal concentrations in the fresh and after catalysis electrolytes (ppm, mg/L), respectively; V_electrolyte_ is the electrolyte volume used (0.025 L for each experiment); and m_metal,catalyst_ is the initial mass of metal in the catalyst (mg).


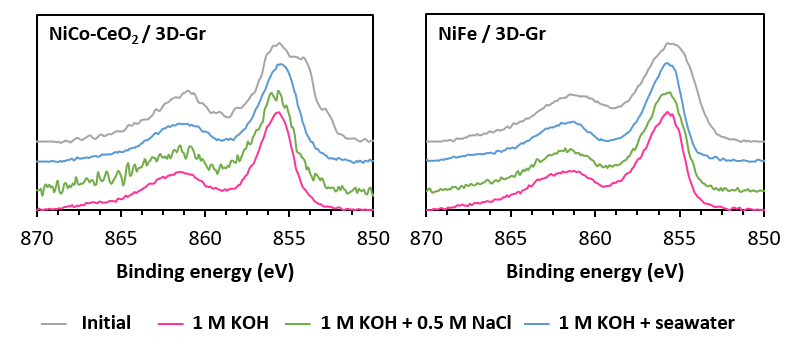


**Figure S18**. High-resolution Ni 2p_3/2_ spectra of NiCo-CeO_2_ / 3D‑Gr and NiFe / 3D‑Gr electrodes recorded after 200 h of overall water splitting in different electrolytes.


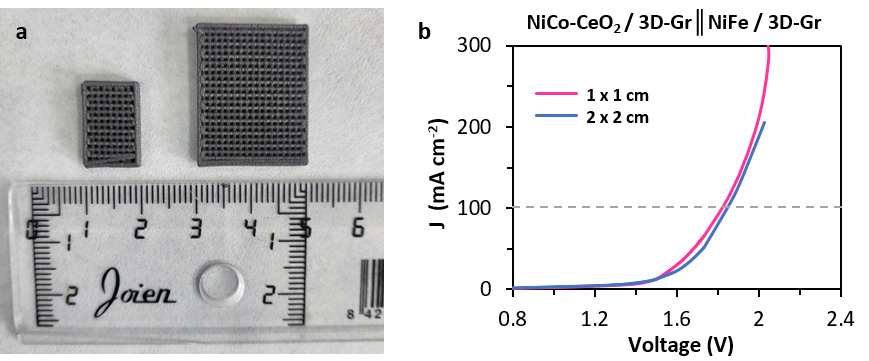


**Figure S19**. Images of the standard and scaled-up 3D-grids, illustrating the increase in electrode dimensions (a). iR-corrected polarization curves of the two-electrode setup with NiCo-CeO_2_ / 3D-Gr || NiFe / 3D-Gr recorded in 1 M KOH at 1 mV s^-1^, comparing the standard and the scaled-up electrodes (b). During the measurements, 85 % iR compensation was applied in the potentiostat. For the comparison shown here, the curves were subsequently corrected ex situ to 100 % to remove the residual uncompensated resistance, which becomes more relevant for the larger electrodes due to the higher absolute currents involved.

**References**

[1] P. Rodríguez Lagar, A. Concheso, D. Barreda, Z. González, M.A. Montes‐Morán, J.A. Menéndez, C. Blanco, R. Santamaría, V.G. Rocha, Direct Ink Writing of 3D‐Structured All‐Carbon Electrodes with High Electrical Conductivity for (Vanadium) Redox Flow Batteries, Advanced Science 12 (2025). https://doi.org/10.1002/advs.202417641.
